# Visual Benefits in Apparent Motion Displays: Automatically Driven Spatial and Temporal Anticipation Are Partially Dissociated

**DOI:** 10.1371/journal.pone.0144082

**Published:** 2015-12-01

**Authors:** Merle-Marie Ahrens, Domenica Veniero, Joachim Gross, Monika Harvey, Gregor Thut

**Affiliations:** 1 School of Psychology, University of Glasgow, Glasgow, United Kingdom; 2 Institute of Neuroscience and Psychology, University of Glasgow, Glasgow, United Kingdom; Centre de Neuroscience Cognitive, FRANCE

## Abstract

Many behaviourally relevant sensory events such as motion stimuli and speech have an intrinsic spatio-temporal structure. This will engage intentional and most likely unintentional (automatic) prediction mechanisms enhancing the perception of upcoming stimuli in the event stream. Here we sought to probe the anticipatory processes that are automatically driven by rhythmic input streams in terms of their spatial and temporal components. To this end, we employed an apparent visual motion paradigm testing the effects of pre-target motion on lateralized visual target discrimination. The motion stimuli either moved towards or away from peripheral target positions (valid vs. invalid spatial motion cueing) at a rhythmic or arrhythmic pace (valid vs. invalid temporal motion cueing). Crucially, we emphasized *automatic* motion-induced anticipatory processes by rendering the motion stimuli non-predictive of upcoming target position (by design) and task-irrelevant (by instruction), and by creating instead endogenous (orthogonal) expectations using symbolic cueing. Our data revealed that the apparent motion cues automatically engaged both spatial and temporal anticipatory processes, but that these processes were dissociated. We further found evidence for lateralisation of anticipatory temporal but not spatial processes. This indicates that distinct mechanisms may drive *automatic* spatial and temporal extrapolation of upcoming events from rhythmic event streams. This contrasts with previous findings that instead suggest an interaction between spatial and temporal attention processes when *endogenously* driven. Our results further highlight the need for isolating intentional from unintentional processes for better understanding the various anticipatory mechanisms engaged in processing behaviourally relevant stimuli with predictable spatio-temporal structure such as motion and speech.

## Introduction

Perception is an active process influenced by attention and expectations. While attention is driven by motivational goals (endogenously) or can be attracted automatically (exogenously), perceptual expectations depend on the history of prior events (or prior knowledge) and consequently on what is most probable regarding forthcoming sensory input (for review see [1]). Anticipatory information is provided across a variety of behaviourally relevant, sensory and cognitive stimuli that generate expectations about forthcoming events through e.g. their inherent temporal and/or spatiotemporal structure. Examples include visual motion [2,3], looming sounds [4,5] and speech stimuli [6–8]. This in turn benefits processing of the future events: Motion stimuli for instance allow predictions of future events in the motion stream both in the spatial and temporal dimensions. Motion stimuli are also effective in capturing attention due to their behavioural relevance (e.g., [9,10]). Accordingly, it is conceivable that motion stimuli engage automatic anticipatory mechanisms that implement an effective, sensory-driven (more reflexive) prediction of forthcoming events. This may occur unintentionally without the need for time-consuming, higher-order cognitive extrapolation of future events, the latter involving intentional analysis and projection of the past motion trajectory to future time points. While there have been many studies on the anticipatory processes linked to spatially and temporally predictive sensory events in the domain of spatial and/or temporal attention (e.g., [11–14]) and apparent motion research (e.g., [15–17]), also dissociating between automatic (unintentional) versus endogenous (intentional) mechanisms (e.g., [18,19]), little is known about the *interaction* between temporal and spatial anticipatory processes, in particular when automatically driven.

Apparent motion cues are discrete events presented at a regular/ rhythmic rate. As a consequence, perception of targets can be probed in or out of the perceived motion path. Several previous studies have investigated the effects of apparent motion stimuli on the processing of such a visual probe [12,15–17,20], with the rationale that due to their predictive structure, apparent motion stimuli may engage perceptually relevant, covert motion completion mechanisms (when eyes fixate). Indeed, such completion mechanisms have been made evident behaviourally [16,17,21]. These completion mechanisms may serve extrapolation as well as interpolation of apparent motion [16] each with likely different perceptual outcomes, namely benefits vs. costs due to anticipation vs. masking effects (for benefits see [12,16,17]; for costs see [15,16,20]). In the present study, we focus on the beneficial effects of motion cueing using a pre-target motion paradigm that, by design, draws on anticipatory (extrapolation) mechanisms [12,22].

In addition to their spatially predictive structure, apparent motion cues also provide predictive information as to the timing of forthcoming events. This can be experimentally explored in isolation by manipulating the temporal structure of static visual flicker stimuli, when no motion is present. Many behavioural studies have shown that rhythmicity *per se* conveys a benefit for target detection at rhythmically cued versus un-cued time points; for instance, when targets are preceded by rhythmic as compared to arrhythmic events [23–25], or when targets are presented in-phase versus out-of-phase in a rhythmic stream of events [12,14,18,26]. Notably, the benefit from rhythmic temporal cueing has been found to be independent of intentionally (endogenously) deployed attention to symbolically cued time points [18]. This suggests that rhythmic stimuli engage automatic anticipatory mechanisms in the temporal dimension (see also [6]).

Finally, research on perceptual benefits from rhythmic cueing has gained momentum from research on its neuronal substrates. Electrophysiological studies have revealed that periodic stimulation leads to phase alignment of ongoing oscillations to the rhythmic input, reflecting entrainment of intrinsic rhythms to the external event streams (e.g., [27–30]). This presumably aligns phases of high neuronal excitability to the expected forthcoming event, a process for which brain oscillations may be ideally placed, given their rhythmic structure [27,28].

In the present behavioural study, we were interested in the beneficial effects of spatial extrapolation as probed by apparent motion and the interaction with temporal anticipation. Importantly, we aimed to investigate for the first time (to the best of our knowledge) how spatial and temporal anticipatory mechanisms are orchestrated when automatically driven. To this end, we presented visual probes in and out of apparent motion trajectories (valid versus invalid spatial trajectory cueing) moving at either rhythmic or arrhythmic pace (valid versus invalid temporal trajectory cueing). We expected this manipulation to enhance perceptual processing for probes appearing at validly motion-cued as compared to invalidly motion-cued time-points and positions, presumably reflecting unintentional, automatic anticipatory mechanisms. However, given that motion stimuli are inherently predictive, it is likely that these types of stimuli also engage higher-order cognitive (intentional) extrapolation processes, unless controlled for, contaminating the automatic extrapolation mechanisms we aimed to study (see [18] for similar arguments regarding stationary flicker). To control for the engagement of automatic versus intentional processes, we kept the motion cues entirely non-predictive of the upcoming target position, and created endogenous (orthogonal) expectations using predictive symbolic cueing instead. In addition, we further emphasized automatic versus intentional processing of motion versus symbolic cues by instruction, explicitly qualifying the motion stimuli as task-irrelevant. This effectively controlled for endogenous engagement of attention to the motion cues, and allowed us to isolate automatic motion-cueing benefits on target detection from endogenous benefits.

## Methods

### Participants

A total of twenty-five healthy participants took part in this study (16 females, 9 males, age range: 19–34, average age ± SD = 23.12 ± 4.21). All participants were right handed and had normal or corrected-to normal vision. Before taking part in the experiment, all participants provided written informed consent. Ethics approval was given by the College of Science and Engineering ethics committee of the University of Glasgow.

### Apparatus

The experiment was presented using E-Prime 2.0 software (Psychology Software Tools, Pittsburgh, PA) on a CRT monitor (Samsung Sync Master 1100MB, 20inch in diameter, spatial resolution of 1280 x 1024 pixels and refresh rate of 85Hz). A chinrest maintained a constant viewing distance of 35cm to the screen. Eye movements were monitored online using a CCTV camera to ensure participants understood the concept of the task (covert attention shifts without eye movements following the cues).

### Stimuli and Task

**Fig 1:**
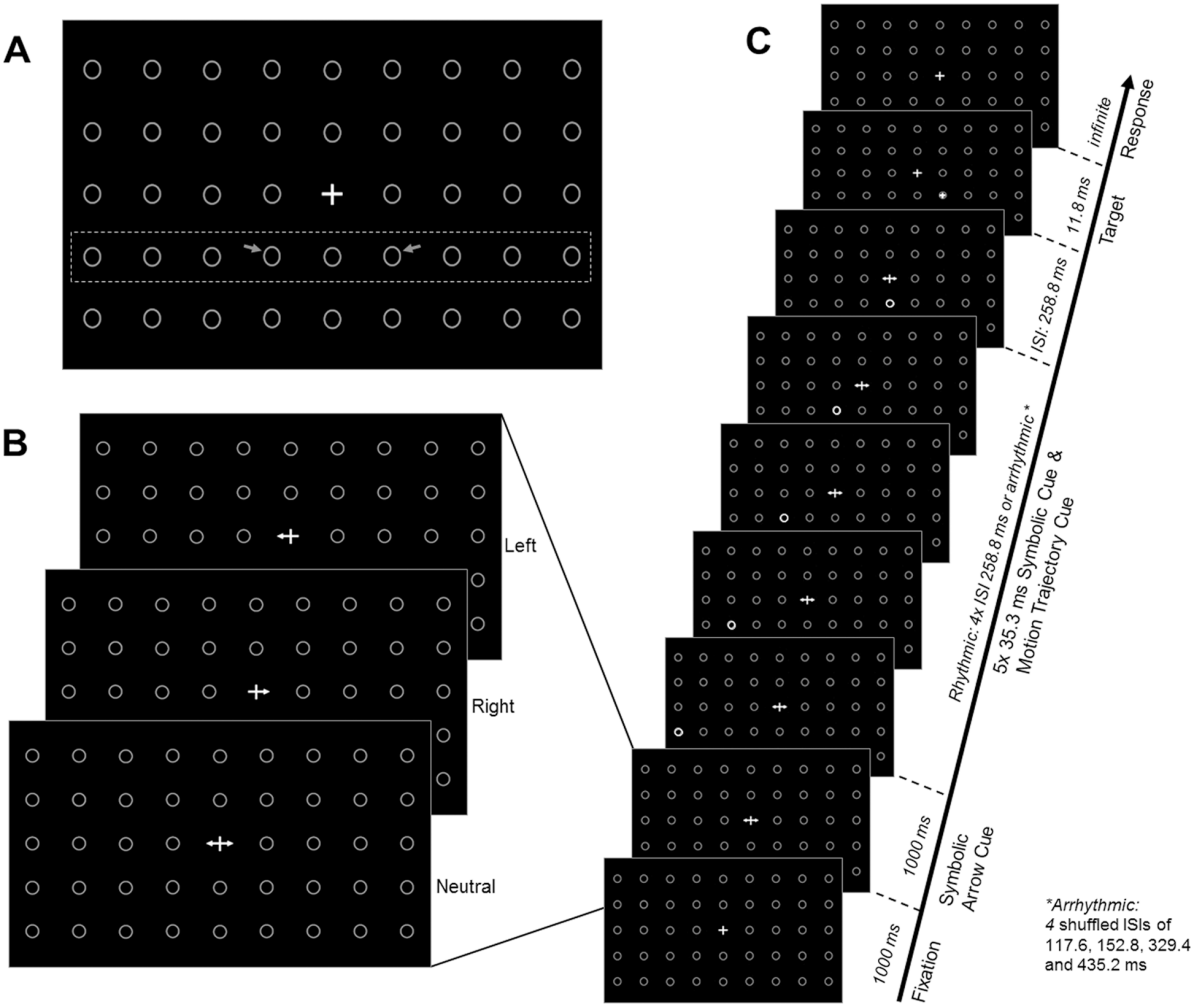
Schematic representation of the experimental design. (A) Fixation cross and placeholders. The dashed rectangle and the arrows (here drawn on top of the background screen for illustrative purposes, not part of visual stimulation) highlight respectively the row in which visual motion cues were presented, and the two possible target locations in the left and right visual fields. (B) Endogenous symbolic arrow-cues (left, right or neutral) as presented in the centre of the screen. (C) Each trial began with a fixation cross (1000ms) and was followed by the presentation of a symbolic cue indicating the probable upcoming target location (here: neutral). The symbolic cue stayed on the screen until target presentation and throughout motion cueing. Motion cueing began 1000ms after symbolic cue onset. Spatial trajectory cueing was implemented by successively flashing (for 35.3ms) each adjacent circle in the row below the symbolic endogenous cue from the left or right periphery towards the centre. Temporal cueing was implemented by presenting the motion cues in either a rhythmic or arrhythmic temporal structure. After a fixed ISI of 1294ms from motion cue onset (including 258.8ms after the last motion cue), the target appeared for 11.8ms either in or out of motion trajectory. Participants were asked to engage in endogenous orienting based on the symbolic cues, and to ignore the motion cues because they were task-irrelevant.

A visual pre-target motion paradigm was implemented (adapted from [22], initially inspired by [12]). A matrix of 5 x 9 circles (placeholders) and a central fixation cross were presented at all times on the screen (Fig 1A). The placeholders were presented in grey on a black background together with the white fixation cross. Symbolic cues presented on top of the fixation cross were all white (Fig 1B). The diameter of the placeholders was 1.2cm, with a vertical distance of 3cm and a horizontal distance of 3.4cm.

The core of a trial consisted of five placeholders in the row below the fixation cross successively flashing from grey to white (left- or rightward x rhythmic or arrhythmic flashes) (Fig 1C). The flashing of the circles either started with the rightmost circle and ended with the central circle directly underneath the fixation cross, or started with the leftmost circle and ended with the same central circle. This created an apparent motion effect of the circles and was followed by a target presented in one of the adjacent placeholders, left or right from the central circle. Thus, targets appeared either in- or out- of apparent motion direction *(for spatial trajectory cueing/ probing spatial extrapolation)*. Left- and rightward motion direction was equally probable and uninformative of upcoming target position (pointing in 50% of trials towards and in the other 50% away from the target).

In addition, the temporal structure of the apparent motion trajectory was manipulated *(for temporal trajectory cueing/ probing temporal extrapolation)*. To this end, the apparent motion cues (flash = 35.3ms) flickered either rhythmically at 3.9Hz (four fixed ISIs of 258.8 ms) or arrhythmically with four intervals of 117.6, 152.8, 329.4 and 435.2 ms (shuffled and presented in random order per arrhythmic trial). These intervals were chosen because visual stimuli moving in discrete steps at these frequencies are perceived as apparent motion [21]. In order to prevent differential forward masking (see also [15]) between rhythmic and arrhythmic conditions, the last interval between the fifth circle and the visual target was fixed at 258.8ms across all trials. In addition, time from motion cue onset to target presentation was fixed across all trials of both the rhythmic and arrhythmic conditions (1294ms). Hence, rhythmic and arrhythmic trials only differed in rhythmic or arrhythmic trial history, but were not differentially informative as to time of target onset, emphasizing differences in deployed unintentional processes (linked to the rhythmicity of motion) rather than endogenous mechanisms (e.g. linked to time-estimation). Rhythmic and arrhythmic trials were presented in random order. Participants were instructed that motion stimuli were uninformative as to both forthcoming target position and time of appearance and therefore irrelevant to the task.

Although motion trajectory were non-predictive as to forthcoming target location and participants were not required to engage with the flicker, participants may still process the apparent motion cues intentionally to extrapolate upcoming events. Thus, to prevent voluntary orienting to the motion cues, participants were asked to engage in a concurrent, symbolically cued endogenous attention orienting task, in anticipation of the upcoming, to-be-discriminated targets: Informative, symbolic arrow-cues were presented at the beginning of the trial, in the centre of the screen (Fig 1), indicating the location of the upcoming target (i.e., left- or rightward arrows, 75% cue-validity) or indicating a neutral trial (bi-directional arrow which was non-predictive (50:50) of target location). Participants were asked to covertly shift attention towards the indicated target position upon presentation of a left- or rightward pointing arrow, or to maintain attention at the fixation cross in neutral trials (and to keep their fixation at the central fixation cross in all cases), while the uninformative motion cues flickered either rhythmically or arrhythmically across the screen (in the background). Targets consisted of a ‘+’ or ‘x’ which needed to be discriminated as fast and accurate as possible by button press.

### Procedure

The experiment took place in two sessions (1 hr per session) on two different days to avoid participants’ fatigue. A training phase familiarized participants with the task. The first training block consisted of intermixed endogenous neutral-, left- or right-cues only (100% validity, number of trials: 24). Participants then completed a second training block, including motion (trajectory cues) in a rhythmic or arrhythmic pattern (50% validity), in addition to the 100% valid endogenous cues (number of trials: 32). This was followed by target titration, which served to individually adjust target luminance contrast to approximately 80% discrimination performance to avoid floor or ceiling effects. Overall, the experiment consisted of three symbolic cues (neutral, left and right), two motion directions (left to right and right to left) and two temporal structures (rhythmic or arrhythmic). All conditions were presented in an intermixed order in five blocks with breaks approximately every 6.5 minutes, resulting in a total number of 960 trials (80 trials per condition) per participant.

### Statistical analysis

We subjected both discrimination accuracy (proportion correct) and reaction times (correct responses only) to two separate fully within-subjects design (repeated-measure) analysis of variances (ANOVAs). The factors of these 3x2x2x2 ANOVAs consisted of Symbolic Spatial Cueing (neutral vs. left vs. right), Spatial Trajectory Cueing (leftward vs rightward motion), Temporal Trajectory Cueing (rhythmic vs. arrhythmic) and Target Location (left vs. right). Significant main effects or interactions were followed up with simple effect tests. Calculation of the effect sizes for simple tests (Cohen’s d) was based on correlated sample comparisons (within-subjects) [31] and we report their magnitude (not the sign).

## Results

**Fig 2:**
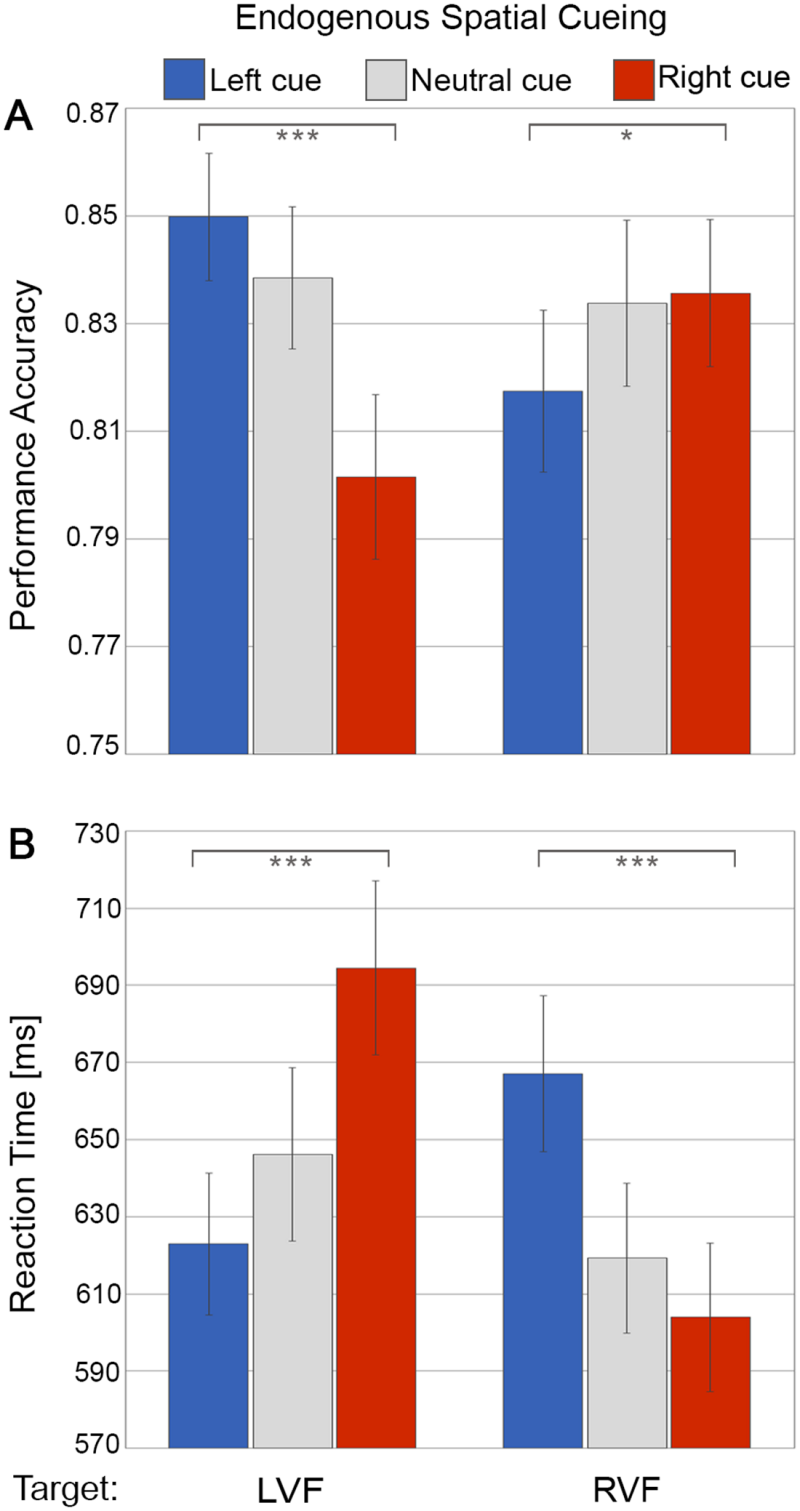
Behavioural results of endogenous spatial cueing. (A) Discrimination accuracy and (B) reaction time. The bar plots represent performance in response to symbolic leftward cues (blue), neutral cues (grey) or rightward cues (red) as a function of target presentation in the left visual field (LVF) versus right visual field (RVF). The error bars indicate standard error of the means (± SE). ‘*’: simple tests significant at p<0.05 and ‘***’ at p < 0.001.

**Fig 3:**
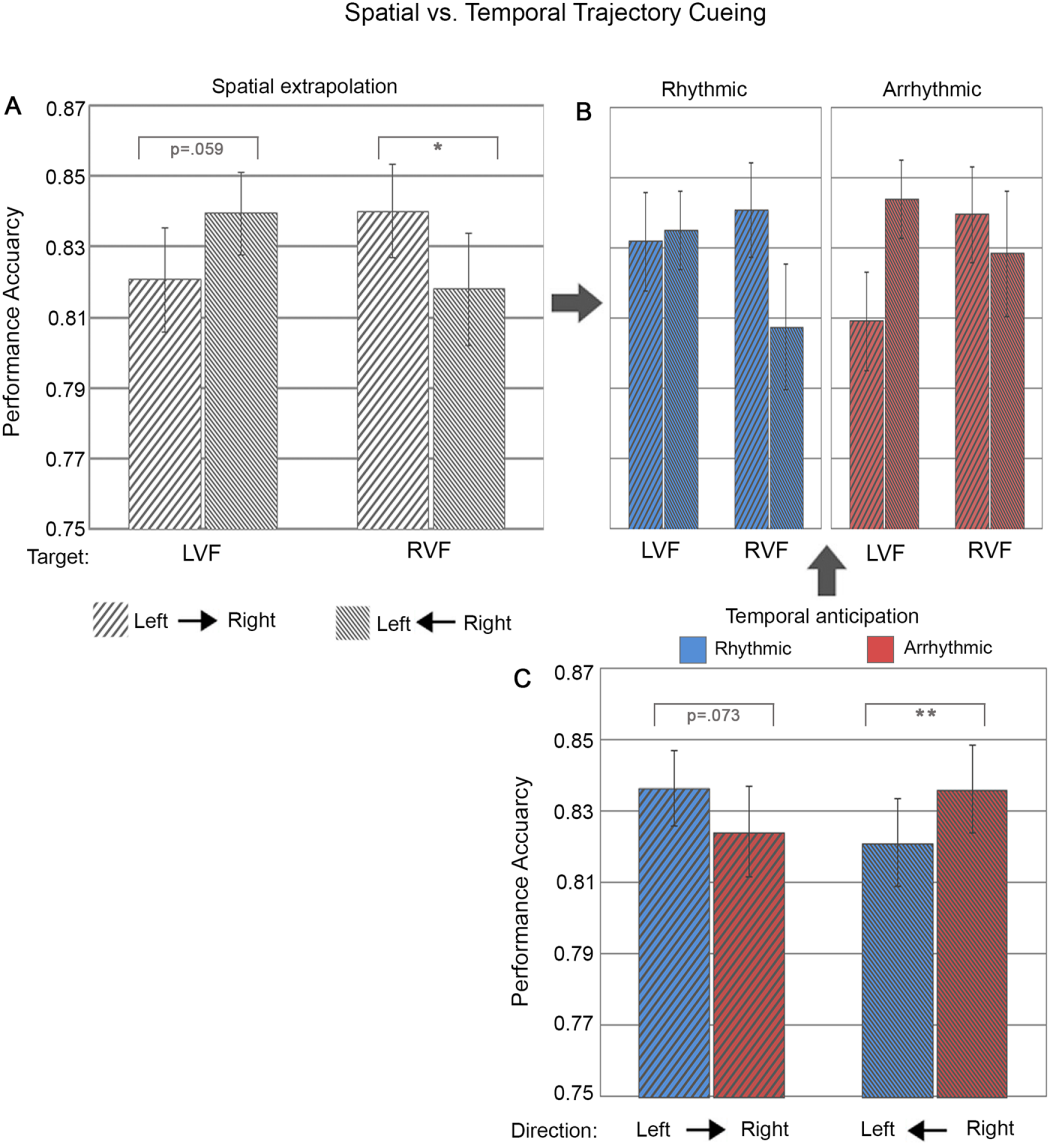
Performance accuracy as a function of spatial trajectory vs. temporal cueing conditions. (A) Bar plots represent performance during right-to-left and left-to-right motion as a function of target locations in the left visual field (LVF) and right visual field (RVF). (B) shows the same as (A) but split between the two levels of temporal cueing (i.e. rhythmic versus arrhythmic cueing). Note that the 2-way interaction of Spatial Trajectory Cueing x Target Location [illustrated in (A)] was statistically independent of temporal trajectory cueing, i.e. there was no significant 3-way interaction [illustrated in (B)]. (C) Separate bar plots for rhythmic (blue) and arrhythmic (red) cueing, per motion cueing direction, illustrating the significant 2-way interaction between temporal trajectory cueing and motion cueing direction. The error bars indicate standard error of the means (± SE). ‘*’: represent simple tests significant at p<0.05 and ‘**’ at p<0.01.

**Fig 4:**
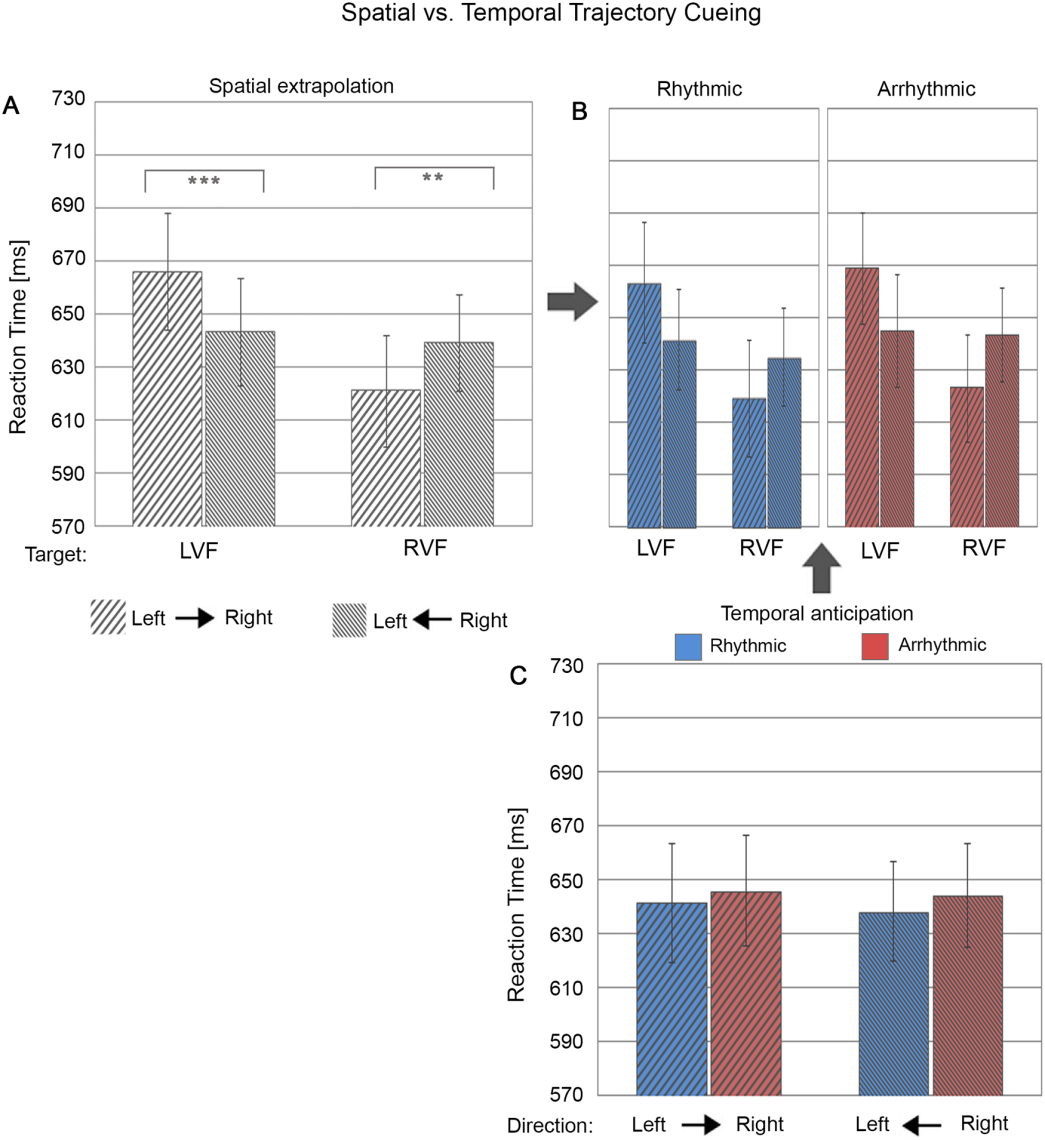
Reaction time as a function of spatial trajectory vs. temporal cueing conditions. (A) Bar plots represent performance during right-to-left and left-to-right motion as a function of target locations in the left visual field (LVF) and right visual field (RVF). (B) shows the same as (A) but split between the two levels of temporal cueing (i.e. rhythmic versus arrhythmic cueing). Note that the 2-way interaction of Spatial Trajectory Cueing x Target Location [illustrated in (A)] was statistically independent of temporal trajectory cueing, i.e. there was no significant 3-way interaction [illustrated in (B)], as for accuracy (see Fig 3). (C) Separate bar plots for rhythmic (blue) and arrhythmic (blue) cueing, per motion direction. The error bars indicate standard error of the means (± SE). ‘**’: represent simple tests significant at p<0.01 and ‘***’ at p<0.001.

The data are represented in Fig 2 (symbolic cueing effects) to Figs 3 and 4 (spatial and temporal trajectory cueing effects and their interactions).

### Endogenous cueing benefit on target discrimination

In line with the participants following the instructions and engaging in the task (endogenous shifts of attention in response to the symbolic cues), we found both discrimination accuracy (Fig 2A) and reaction time (Fig 2B) to be influenced by endogenous cueing direction (left, neutral, right symbolic cues) as a function of target position (left visual field vs right visual field), which showed in a significant 2-way interaction of Endogenous Spatial Cueing x Target Location both for accuracy (F(2,48) = 13.32, p = .00003, *ηp* ^*2*^ = .36) and reaction time (F(2, 48) = 83.08, p < .00001, *ηp* ^*2*^ = .78). Follow-up simple tests showed significantly better performance levels (higher accuracy, faster RTs) for validly than invalidly cued targets in both the left visual field (accuracy L- vs R-cue: F(1,24) = 21.79, p = .00001, Cohen’s d = .93; RT L- vs R-cue: F(1,24) = 80.18, p < .00001, Cohen’s d = 1.79), and the right visual field (accuracy R- vs. L-cue: F(1,24) = 5.05, p = .034, Cohen’s d = .45; RT R- vs. L-cue: F(1,24) = 66.56, p < .00001, Cohen’s d = 1.63).

Importantly, there was no evidence for motion to affect any of the above endogenous cueing benefits at attended locations (no 3 way interaction of Symbolic Spatial Cueing x Target Location with neither Spatial Trajectory Cueing nor Temporal Trajectory Cueing, for any of the two measures (accuracy and RT) (both 3-way interactions non-significant: F(2,48) < 0.44, p > .646, *ηp* ^*2*^ < .02). This speaks in favour of the participants maintaining endogenous attention throughout all conditions independently of the presence of simultaneous motion cues, i.e. for participants not dividing endogenous attention between the symbolic endogenous and the motion cues. Or in other words, this shows that participants effectively ignored the motion cues, as desired by instructions and design (motion flicker task-irrelevant and non-predictive as to forthcoming target location). As a consequence, any benefit from spatial or temporal trajectory cueing can be considered to reflect automatic anticipation.

Finally but tangential to the questions of this study, the overall ANOVAs revealed a main effect of Endogenous Cueing for both performance accuracy (F(2,48) = 5.92, p = .005, *ηp* ^*2*^ = .20) and reaction time (F(2, 48) = 13.88, p = .00002, *ηp* ^*2*^ = .37).

### Automatic spatial extrapolation in response to the motion trajectory: Benefits of motion direction on target discrimination

Despite being task-irrelevant and non-predictive, the direction of motion trajectory (leftward vs. rightward motion) significantly affected both target discrimination accuracy (Fig 3A) and reaction time (Fig 4A) as a function of target location (LVF vs. RVF), as revealed by significant 2-way interactions of Spatial Trajectory Cueing (i.e. motion direction) x Target Location (accuracy: F(1,24) = 10.14, p = .004, *ηp* ^*2*^ = .30; RT F(1, 24) = 50.93, p < .00001, *ηp* ^*2*^ = .68). Follow-up simple tests revealed significantly (or near-significantly) better performance levels (higher accuracy, faster RTs) for validly as compared to invalidly cued targets for both the right visual field (accuracy right- vs. leftward motion: F(1,24) = 6.80, p = .015, Cohen’s d = .52/ RT right- vs. leftward motion: F(1,24) = 10.18, p = .004, Cohen’s d = .64) and the left visual field (accuracy left vs. rightward motion: F(1,24) = 3.94, p = .059, Cohen’s d = .40; RT left vs. rightward motion: F(1,24) = 31.22, p = .00001, Cohen’s d = 1.12). Thus, motion direction clearly benefitted target discrimination at motion-cued locations, with higher performance accuracy and faster reaction times for targets appearing in as compared to out of motion trajectory. This is evidence for motion trajectory automatically driving spatial anticipation. Interestingly, this effect was independent of temporal trajectory cueing (rhythmicity) for both accuracy (F(1,24) = .18, p = .67, *ηp* ^*2*^ = .008) and reaction time (F(1, 24) = .26, p = .62, *ηp* ^*2*^ = .01) (see Figs 3B and 4B).

Finally and again tangential to our question, there was a main effect of target location, with faster responses for targets in the right visual field relative to the left visual field (difference of 24.46 ms) (F(1,24) = 12.38, p = .002, *ηp* ^*2*^ = .34).

### Automatic temporal anticipation in response to the motion trajectory: Benefits from the temporal structure of motion stimuli

Overall responses to rhythmic cueing were slightly faster relative to arrhythmic motion cues (difference of 5.3 ms) (F(1, 24) = 4.72, p = .034, *ηp* ^*2*^ = .16). More importantly temporal cueing (rhythmicity) influenced target discrimination but differently from spatial trajectory cueing. We expected that the benefit from automatically driving spatial anticipation by motion (as revealed above in the 2-way interaction Spatial Trajectory Cueing x Target Location) would be enhanced by temporal cueing (rhythmic vs arrhythmic condition), which was however not the case (no 3-way interaction of Temporal Cueing x Spatial Trajectory Cueing x Target Location, see above). Instead, temporal trajectory cueing benefits were limited to discrimination accuracy and depended on motion direction (significant 2-way interaction of Temporal Trajectory Cueing x Spatial Trajectory Cueing (F(1,24) = 16.16, p = .0005, *ηp* ^*2*^ = .40)(Fig 3C). This effect was absent for reaction times (Fig 4C). Follow-up simple tests on performance accuracy showed a trend for better performance when exposed to rhythmic as compared to arrhythmic rightward motion (F(1,24) = 3.53, p = .073, Cohen’s d = .38) and a significant advantage for arrhythmic as compared to rhythmic leftward motion (F(1,24) = 10.01, p = .004, Cohen’s d = .63). This finding indicates asymmetric effects of temporal trajectory cueing (rhythmic vs. arrhythmic) for cueing towards the right vs. left visual fields.

## Discussion

We here tested the interaction between automatically driven temporal and spatial anticipatory processes in response to apparent motion stimuli by testing the effects of pre-target motion on target discrimination in or out of the motion path, at a rhythmic or arrhythmic rate. To isolate the effects of automatic processes putatively driven by the motion stream from those of intentional engagement of attention to the motion cues (i.e. endogenous attentional confounds), we asked participants to consider motion as task-irrelevant and to engage instead in an endogenous (and orthogonal) attention task. Our data revealed that this effectively avoided engagement of endogenous attention to the motion cues, given that motion did not affect the benefits of target perception at the focus of endogenous attention (no evidence for divided endogenous attention between the task and the motion stimuli). We therefore interpret the effects of apparent motion on target processing in the context of automatically driven processes.

Our main findings were three fold. First, we found that pre-target motion cues conveyed a benefit for target processing at spatially cued versus un-cued locations in terms of both accuracy and reaction time. These benefits were however not influenced by the presence or absence of temporally valid cueing, here rhythmic or arrhythmic motion streams. This indicates that the inherently predictive spatial structure of motion automatically facilitated perception at forthcoming locations along the motion trajectory, yet without strict temporal constraints. Second, we found that the temporal structures of the apparent motion stream conveyed perceptual benefits for target processing. While these perceptual benefits were independent of the presence of spatially valid motion cues, they depended on the direction of motion suggesting hemispheric lateralization. This indicates that spatial and temporal anticipatory processes in response to regular vs. irregular motion streams follows distinct rules by which visual perception is facilitated. Third, our finding that motion stimuli did not influence the effects of endogenous (orthogonal) expectations created by symbolic cueing suggests that automatically driven anticipatory processes can be independent from intentionally driven (higher-order) processes.

This corroborates and extends previous research on entrainment of anticipatory processes by natural stimuli (such as motion) with a spatiotemporally predictive structure, which by definition convey behavioural relevance. Our findings support the notion that anticipatory sensory processes, while strongly influenced by internal goals likely involving higher level top-down attentional mechanisms [27], can also be automatically driven in the presence of external events [32,33]. Importantly, here we reveal for the first time the orchestration of automatic spatial and temporal anticipatory processes, and show that these processes originate from partially distinct mechanisms (when investigated with behavioural measures). This possibly occurs bottom-up without the recruitment of higher level cognitive resources (see also [18]). Apart from the processing of motion stimuli, such mechanisms may be engaged in speech communication, comprehension and attention, where timing is crucial for predicting internalized regularities of events (for reviews see [8,34]). Below, we discuss the mechanisms that may underlie automatic spatial and temporal anticipation and their relation in light of research on apparent motion, entrainment and attentional cueing.

### Spatial extrapolation automatically driven by apparent motion stimuli

We found that perceptual processing was clearly enhanced when targets appeared at spatially extrapolated locations in the motion direction, despite the fact that the apparent motion cues were task-irrelevant and non-predictive. This extends prior studies showing perceptual benefits when employing apparent motion stimuli (and also with attentive and/or passive object tracking paradigms), in which the observers traced (covertly) an object while perception of a target was probed in or out of the object’s motion path (e.g., [12,16,17,22]). These perceptual benefits are likely conveyed through mechanisms for maintaining and updating the representation of a moving object along an apparent motion trajectory, serving motion extrapolation [16] and interpolation [16,17,21]. In apparent motion this occurs even outside of the voluntary attentional focus [16]. As an explanatory mechanism for the perceptual benefits, smooth shifts in the attentional focus along the motion path have been suggested, tracking the moving object and predicting future target locations [17,21,35]. This could either be mediated by conscious prediction or an internal model [21], updating the motion path intentionally or automatically. Alternatively, low-level motion processing could explain motion prediction mechanisms. As proposed by Nijhawan [36,37], early visual structures may compensate for neuronal processing delays through extrapolation, attempting to predict future locations of a moving object. Our present findings of spatial motion trajectory facilitating perception provide support for automatic prediction mechanisms. This is also in line with prior findings showing that contrast sensitivity to moving objects is enhanced towards the end of the motion trajectory, interpreted to reflect automatic attention capture and prediction mechanisms [38], and that motion induces a forward prediction signal [39].

### Temporal anticipation automatically driven by rhythmic (versus arrhythmic) apparent motion stimuli is partially independent from spatial extrapolation

We found perception to be modulated by temporal trajectory cueing. Interestingly however, effects of temporal cueing were independent of spatial cueing, i.e. we did not find any synergy/additive effects of temporal (rhythmic) cueing on the spatial cueing benefit with our design. Instead, temporal cueing showed an unexpected pattern (not observed with spatial cueing): it depended on motion direction. That is, rhythmic cueing tended to benefit perceptual processing with rightward motion (relative to arrhythmic cueing), while arrhythmic cueing was associated with better performance in response to leftward motion (as compared to rhythmic cueing). This asymmetrical perceptual benefit driven by temporal cueing may suggest that distinct mechanisms are at play for spatial and temporal prediction. Importantly, their independence implies that these mechanisms do not interact in our design, i.e. at a purely unintentional/ automatic level (when endogenous spatial attention is saturated).

In contrast to our results, a recent series of studies that concurrently manipulated spatial and temporal attention have shown that temporal attention on its own is not effective in modulating visual performance [13] nor in modulating early visual evoked potentials typically associated with spatial attention [12]. Instead, these studies provide evidence for synergistic effects of temporal on spatial attention, i.e. for the need of spatial processes to be engaged so that temporal advantages can be expressed [12,13]. However, these results are not directly comparable to our findings because of differences in experimental design. Rohenkohl et al. [13] manipulated temporal and spatial expectations in the endogenous dimension. Doherty et al. [12] used apparent motion with an intrinsic, spatially and temporally predictive structure but did not control for intentional deployment of attention to these cues. As a consequence, participants may have engaged in endogenous anticipatory processes to deliberately use the apparent motion information for intentionally predicting the forthcoming events. Hence, synergistic interaction between these systems may require endogenous control to be expressed. Similar to our results, Jones [14] found temporal and spatial cueing to convey independent attentional benefits. However, Jones [14] studied the interaction between endogenous spatial attention and exogenous temporal expectations using symbolic spatial and central flicker cues, i.e. crossing the endogenous/ exogenous divide, again limiting comparison to our results. The discrepancy between our own and previous findings hence suggests that anticipatory processes in the spatial and temporal dimension may differ as to whether the cue is rhythmic or symbolic, as previously suggested [11,13,18,23,40,41], as well as whether the processes reflect intentional versus automatic mechanisms, as suggested here.

### Possible neuronal substrates of dissociated automatic spatial and temporal anticipation with apparent motion stimuli

It is well established that the left hemisphere is dominant for processing temporal information, whereas the right hemisphere is more specialized in processing spatial information [42–45]. In line with this view, previous studies have associated temporal attention with left hemispheric activity [11,12]. Coull and Nobre [11], for example, observed left intraparietal and premotor cortex activity for temporal orienting (i.e. in areas engaged in motor planning and attention; [46]) versus right intraparietal activity for spatial orienting. Doherty et al. [12], who found temporal attention to be associated with motor response-related EEG components, again interpreted this to reflect the engagement of left hemispheric resources with temporal attention deployment (in line with [11]). Our finding of a left-right asymmetry showing that perception tends to be enhanced by rhythmic (as compared to arrhythmic) motion but only for the rightward motion cues may hence suggest for the first time that hemispheric (left lateralized) differences may also come into play for automatic temporal anticipatory mechanisms. By extension, our finding that perception was enhanced for arrhythmic (as compared to rhythmic) leftward motion may suggest a right hemispheric process. However, these findings on asymmetry should be interpreted with caution given that they were unexpected. In this light, it is of interest to note that most previous studies reporting perceptual benefits from temporal cueing with pre-target motion paradigms only employed rightward motion (albeit for testing more endogenous attention) [12,23,47]. One exception is De Graaf et al. [22], who used a symmetric design (including left and rightward motion) but did not report an asymmetrical benefit in favour of rhythmic rightward motion (note that no arrhythmic condition was tested). However, the results of De Graaf et al [22] are likely to be confounded by intentional prediction mechanisms, which were not controlled for by design in contrast to the present study. Hence, comparisons to prior studies are limited and follow-up experiments are needed to confirm the hemifield asymmetry we found.

For a possible explanation of the observed hemifield asymmetry, we speculate that the entrainment of the attention focus to rhythmic cues may draw on similar resources as entrainment to rhythmic (and therefore predictive) speech signals (for review see [8]), for which a left hemispheric dominance, albeit not exclusively, can be assumed [48,49]. Alternatively, directional preferences for rightward motion stimuli [50, 51], or a larger rightward shift of attention for rightward (but not leftward) motion [52], may be due to internalized/learned reading habits and thus preferential visual rightward scanning. However, such a bias should be observed not only for temporal but also spatial processes, which was not the case here. Indeed, research on participants with native languages read/written from left to right (e.g. English) has shown that their perceptual span is asymmetrically shifted to the right around the fixation point [53], while this effect is reversed for participants with native languages read/written from right to left [54,55]. Thus, a bias from reading habits is unlikely to explain the dissociation we observe here between temporal and spatial anticipatory processes.

## Conclusion

Prior findings suggest synergistic effects of endogenous temporal and spatial expectations [11–13]. In contrast, we here controlled for higher level (top-down) processes and found evidence for behaviourally dissociated processes of temporal and spatial anticipation when automatically driven by motion stimuli. This establishes differences between endogenously and automatically driven anticipatory mechanisms in response to predictive stimuli. Follow-up studies employing neuroimaging could help to better understand whether the behavioural dissociation revealed here is also reflected in separate neurophysiological signatures. We conclude that it is important to control for the various levels of anticipatory processes (intentional vs. automatic) in order to better understand the interplay between the various top-down and bottom-up mechanisms of sensory prediction, and their effects on perception.

## Supporting Information

S1 Data Spreadsheet containing individual participant data. Sheet 1: Performance accuracy. Sheet 2: Reaction time.
(XLSX)

## References

[1] Summerfield C, Egner T. Expectation (and attention) in visual cognition. Trends Cogn Sci. 2009;13(9):403–9. 10.1016/j.tics.2009.06.003 19716752

[2] Adelson EH, Bergen JR. Spatiotemporal energy models for the perception of motion. J Opt Soc Am A. 1985;2(2):284–99. 3973762 10.1364/josaa.2.000284

[3] Khoei M a., Masson GS, Perrinet LU. Motion-based prediction explains the role of tracking in motion extrapolation. J Physiol Paris. Elsevier Ltd; 2013;107(5):409–20. 10.1016/j.jphysparis.2013.08.001 24036184

[4] Rosenblum LD, Wuestefeld AP, Saldaña HM. Auditory looming perception: influences on anticipatory judgments. Perception. 1993;22(12):1467–82. 8090622 10.1068/p221467

[5] Ghazanfar AA, Neuhoff JG, Logothetis NK. Auditory looming perception in rhesus monkeys. Proc Natl Acad Sci U S A. 2002;99(24):15755–7. 12429855 10.1073/pnas.242469699 PMC137788

[6] Jones MR, Boltz M. Dynamic attending and responses to time. Psychol Rev. 1989;96(3):459–91. 2756068 10.1037/0033-295x.96.3.459

[7] Zion Golumbic EM, Poeppel D, Schroeder CE. Temporal context in speech processing and attentional stream selection: a behavioral and neural perspective. Brain Lang. 2012;122(3):151–61. 10.1016/j.bandl.2011.12.010 22285024 PMC3340429

[8] Arnal LH, Giraud AL. Cortical oscillations and sensory predictions. Trends Cogn Sci. 2012;16(7):390–8. 10.1016/j.tics.2012.05.003 22682813

[9] Franconeri SL, Simons DJ. Moving and looming stimuli capture attention. Percept Psychophys. 2003;65(7):999–1010. 14674628 10.3758/bf03194829

[10] Al-Aidroos N, Guo RM, Pratt J. You can’t stop new motion: Attentional capture despite a control set for colour. Vis cogn. 2010;18(6):859–80.

[11] Coull JT, Nobre AC. Where and when to pay attention: the neural systems for directing attention to spatial locations and to time intervals as revealed by both PET and fMRI. J Neurosci. 1998;18(18):7426–35. 9736662 10.1523/JNEUROSCI.18-18-07426.1998 PMC6793260

[12] Doherty JR, Rao A, Mesulam MM, Nobre AC. Synergistic effect of combined temporal and spatial expectations on visual attention. J Neurosci. 2005;25(36):8259–66. 16148233 10.1523/JNEUROSCI.1821-05.2005 PMC6725546

[13] Rohenkohl G, Gould IC, Pessoa J, Nobre AC. Combining spatial and temporal expectations to improve visual perception. J Vis. 2014;14(4):1–13. 10.1167/14.4.8 PMC3983934 24722562

[14] Jones A. Independent effects of bottom-up temporal expectancy and top-down spatial attention. An audiovisual study using rhythmic cueing. Front Integr Neurosci. Frontiers; 2015;8:96. 10.3389/fnint.2014.00096 PMC4285055 25610378

[15] Schwiedrzik CM, Alink A, Kohler A, Singer W, Muckli L. A spatio-temporal interaction on the apparent motion trace. Vision Res. 2007;47:3424–33. 18053847 10.1016/j.visres.2007.10.004

[16] Hogendoorn H, Carlson T a., Verstraten F a J. Interpolation and extrapolation on the path of apparent motion. Vision Res. 2008;48(7):872–81. 10.1016/j.visres.2007.12.019 18279906

[17] Shioiri S, Yamamoto K, Kageyama Y, Yaguchi H. Smooth shifts of visual attention. Vision Res. 2002;42(26):2811–6. 12450506 10.1016/s0042-6989(02)00405-4

[18] Breska A, Deouell LY. Automatic Bias of Temporal Expectations following Temporally Regular Input Independently of High-level Temporal Expectation. J Cogn Neurosci. 2014;26(7):1555–71. 10.1162/jocn_a_00564 24392898

[19] Olk B. Effects of spatial, temporal and spatiotemporal cueing are alike when attention is directed voluntarily. Exp Brain Res. 2014;232(11):3623–33. 10.1007/s00221-014-4033-7 25081102

[20] Yantis S, Nakama T. Visual interactions in the path of apparent motion. Nat Neurosci. 1998;1(6):508–12. 10196549 10.1038/2226

[21] Shioiri S, Cavanagh P, Miyamoto T, Yaguchi H. Tracking the apparent location of targets in interpolated motion. Vision Res. 2000;40(10–12):1365–76. 10788646 10.1016/s0042-6989(99)00249-7

[22] De Graaf T a, Gross J, Paterson G, Rusch T, Sack AT, Thut G. Alpha-band rhythms in visual task performance: phase-locking by rhythmic sensory stimulation. PLoS One. 2013;8(3):e60035 10.1371/journal.pone.0060035 23555873 PMC3612058

[23] Rohenkohl G, Coull JT, Nobre AC. Behavioural dissociation between exogenous and endogenous temporal orienting of attention. PLoS One. 2011;6(1):1–5. 10.1371/journal.pone.0014620 PMC3030556 21297968

[24] Rohenkohl G, Cravo AM, Wyart V, Nobre AC. Temporal Expectation Improves the Quality of Sensory Information. J Neurosci. 2012;32(24):8424–8. 10.1523/JNEUROSCI.0804-12.2012 22699922 PMC4235252

[25] Cravo AM, Rohenkohl G, Wyart V, Nobre AC. Temporal expectation enhances contrast sensitivity by phase entrainment of low-frequency oscillations in visual cortex. J Neurosci. 2013;33(9):4002–10. 10.1523/JNEUROSCI.4675-12.2013 23447609 PMC3638366

[26] Mathewson KE, Fabiani M, Gratton G, Beck DM, Lleras A. Rescuing stimuli from invisibility: Inducing a momentary release from visual masking with pre-target entrainment. Cognition. 2010;115(1):186–91. 10.1016/j.cognition.2009.11.010 20035933

[27] Lakatos P, Karmos G, Mehta AD, Ulbert I, Schroeder CE. Entrainment of neuronal oscillations as a mechanism of attentional selection. Science. 2008;320(5872):110–3. 10.1126/science.1154735 18388295

[28] Schroeder CE, Lakatos P. Low-frequency neuronal oscillations as instruments of sensory selection. Trends Neurosci. 2009;32(1):9–18. 10.1016/j.tins.2008.09.012 19012975 PMC2990947

[29] Mathewson KE, Prudhomme C, Fabiani M, Beck DM, Lleras A, Gratton G. Making waves in the stream of consciousness: entraining oscillations in EEG alpha and fluctuations in visual awareness with rhythmic visual stimulation. J Cogn Neurosci. 2012;24(12):2321–33. 10.1162/jocn_a_00288 22905825

[30] Spaak E, de Lange FP, Jensen O. Local entrainment of α oscillations by visual stimuli causes cyclic modulation of perception. J Neurosci. 2014;34(10):3536–44. 10.1523/JNEUROSCI.4385-13.2014 24599454 PMC6608988

[31] Lakens D. Calculating and reporting effect sizes to facilitate cumulative science: a practical primer for t-tests and ANOVAs. Front Psychol. 2013;4:863 10.3389/fpsyg.2013.00863 24324449 PMC3840331

[32] Large EW, Jones MR. The Dynamics of Attending: How People Track Time-Varying Events. Psychol Rev. 1999;106(1):119–59.

[33] Jones MR, Moynihan H, MacKenzie N, Puente J. Temporal aspects of stimulus-driven attending in dynamic arrays. Psychol Sci. 2002;13(4):313–9. 12137133 10.1111/1467-9280.00458

[34] Calderone DJ, Lakatos P, Butler PD, Castellanos FX. Entrainment of neural oscillations as a modifiable substrate of attention. Trends Cogn Sci. 2014;18(6):300–9. 10.1016/j.tics.2014.02.005 24630166 PMC4037370

[35] Cavanagh P. Attention-Based Motion Perception. Science (80-). 1992;257:1563–5. 10.1126/science.1523411 1523411

[36] Nijhawan R. Motion extrapolation in catching. Nature. 1994;370. 10.1038/370256b0 8035873

[37] Khurana B, Nijhawan R. Extrapolation or attention shift? Nature. 1995;378. 10.1038/378565a0 8524389

[38] Verghese P, McKee SP. Predicting future motion. J Vis. 2002;2(5):413–23. 12678655 10.1167/2.5.5

[39] Roach NW, McGraw P V., Johnston A. Visual motion induces a forward prediction of spatial pattern. Curr Biol. Elsevier Ltd; 2011;21(9):740–5. 10.1016/j.cub.2011.03.031 PMC3093611 21514158

[40] Triviño M, Correa Á, Arnedo M, Lupiáñez J. Temporal orienting deficit after prefrontal damage. Brain. 2010;133(4):1173–85. 20145048 10.1093/brain/awp346

[41] Triviño M, Arnedo M, Lupiáñez J, Chirivella J, Correa Á. Rhythms can overcome temporal orienting deficit after right frontal damage. Neuropsychologia. 2011;49(14):3917–30. 10.1016/j.neuropsychologia.2011.10.009 22019698

[42] Bradshaw JL, Nettleton NC. The nature of hemispheric specialization in man. Behav Brain Sci. 1981;4(01):51.

[43] Hammond G. Hemsipheric Differences in Temporal Resolution. Brain Cogn. 1982; 10.1016/0278-2626(82)90009-4 6765473

[44] Nicholls ME. Temporal processing asymmetries between the cerebral hemispheres: evidence and implications. Laterality. 1996;1(2):97–137. 15513031 10.1080/713754234

[45] Kinsbourne M. Hemi-neglect and hemisphere rivalry. Adv Neurol. 1977;18:41–9. 920524

[46] Rushworth MF, Krams M, Passingham RE. The attentional role of the left parietal cortex: the distinct lateralization and localization of motor attention in the human brain. J Cogn Neurosci. 2001;13(5):698–710. 11506665 10.1162/089892901750363244

[47] Rohenkohl G, Nobre AC. Alpha Oscillations Related to Anticipatory Attention Follow Temporal Expectations. J Neurosci. 2011;31(40):14076–84. 10.1523/JNEUROSCI.3387-11.2011 21976492 PMC4235253

[48] Gross J, Hoogenboom N, Thut G, Schyns P, Panzeri S, Belin P, et al Speech rhythms and multiplexed oscillatory sensory coding in the human brain. PLoS Biol. 2013;11(12):e1001752 10.1371/journal.pbio.1001752 24391472 PMC3876971

[49] Park H, Ince RAA, Schyns PG, Thut G, Gross J. Frontal Top-Down Signals Increase Coupling of Auditory Low-Frequency Oscillations to Continuous Speech in Human Listeners. Curr Biol. The Authors; 2015;25(12):1649–53. 10.1016/j.cub.2015.04.049 PMC4503802 26028433

[50] Halpern a R, Kelly MH. Memory biases in left versus right implied motion. J Exp Psychol Learn Mem Cogn. 1993;19(2):471–84. 8454967 10.1037//0278-7393.19.2.471

[51] Müller HJ, von Mühlenen A. Attentional tracking and inhibition of return in dynamic displays. Percept Psychophys. 1996;58(2):224–49. 8838166 10.3758/bf03211877

[52] Kerzel D. Attention maintains mental extrapolation of target position: Irrelevant distractors eliminate forward displacement after implied motion. Cognition. 2003;88:109–31. 12711155 10.1016/s0010-0277(03)00018-0

[53] Rayner K, Well AD, Pollatsek a. Asymmetry of the effective visual field in reading. Percept Psychophys. 1980;27(6):537–44. 7393701 10.3758/bf03198682

[54] Pollatsek a, Bolozky S, Well AD, Rayner K. Asymmetries in the perceptual span for Israeli readers. Brain Lang. 1981;14(1):174–80. 7272722 10.1016/0093-934x(81)90073-0

[55] Nachshon I. Directional preferences in perception of visual stimuli. Int J Neurosci. 1985;25(3–4):161–74. 3884523 10.3109/00207458508985369

